# *N*-Acetylneuraminic acid attenuates hypercoagulation on high fat diet-induced hyperlipidemic rats

**DOI:** 10.3402/fnr.v59.29046

**Published:** 2015-12-04

**Authors:** Zhang Yida, Mustapha Umar Imam, Maznah Ismail, WaiTeng Wong, Maizaton Atmadini Abdullah, Aini Ideris, Norsharina Ismail

**Affiliations:** 1 Laboratory of Molecular Biomedicine, Institute of Bioscience, Universiti Putra Malaysia, Serdang, Malaysia; 2 Cardiology Department, Affiliated Hospital of Chengde Medical University, Chengde, China; 3 Department of Nutrition and Dietetics, Faculty of Medicine and Health Sciences, Universiti Putra Malaysia, Serdang, Malaysia; 4 Department of Pathology, Faculty of Medicine and Health Sciences, Universiti Putra Malaysia, Serdang, Malaysia; 5 Faculty of Veterinary Medicine, Universiti Putra Malaysia, Serdang, Malaysia

**Keywords:** high fat diet, coagulation, hypercholesterolemia, *N*-acetylneuraminic acid, sialic acid

## Abstract

**Background and objective:**

*N*-Acetylneuraminic acid (Neu5Ac), a type of sialic acid, has close links with cholesterol metabolism and is often used as a biomarker in evaluating the risk of cardiovascular diseases. However, most studies on the health implications of Neu5Ac have focused on its effects on the nervous system, while its effects on cardiovascular risk factors have largely been unreported. Thus, the effects of Neu5Ac on coagulation status in high fat diet (HFD)-induced hyperlipidemic rats were evaluated in this study.

**Methods:**

Sprague Dawley male rats were divided into five different groups and fed with HFD alone, HFD low-dose Neu5Ac, HFD high-dose Neu5Ac, HFD simvastatin (10 mg/kg day), and normal pellet alone. Food was given *ad libitum* while body weight of rats was measured weekly. After 12 weeks of intervention, rats were sacrificed and serum and tissue samples were collected for biochemistry and gene expression analysis, respectively.

**Results:**

The results showed that Neu5Ac could improve lipid metabolism and hyperlipidemia-associated coagulation. Neu5Ac exerted comparable or sometimes better physiological effects than simvastatin, at biochemical and gene expression levels.

**Conclusions:**

The data indicated that Neu5Ac prevented HFD-induced hyperlipidemia and associated hypercoagulation in rats through regulation of lipid-related and coagulation-related genes and, by extension, induced metabolite and protein changes. The implications of the present findings are that Neu5Ac may be used to prevent coagulation-related cardiovascular events in hyperlipidemic conditions. These findings are worth studying further.

Sialic acids (SA) work as modulators in both molecular and cellular interactions and also mediate functional roles in health and diseases (1, 2). *N*-Acetylneuraminic acid (Neu5Ac) and *n*-glycolylneuraminic acid (Neu5Gc) are the predominant forms of SAs in mammals, although Neu5Gc, which is the non-human form of SA, has been associated with harmful effects when consumed (3, 4). Additionally, serum SA level is an important clinical indicator in disease diagnosis, especially in coronary artery disease (5, 6). Accordingly, high levels of serum SA in combination with dyslipidemia have been associated with cardiovascular diseases (7, 8). It is an acute response marker which is believed to indicate cardiovascular disease-associated inflammation (9). However, there are indications that elevated SA levels in cardiovascular disease may be a counter mechanism to reverse atherosclerosis, through the use of the SA as a substrate for the resialylation of vascular endothelium (10). SA is richly found in vascular endothelium and erythrocyte. This negatively charged molecule can inhibit blood clotting and possibly prevent clinical manifestations of cardiovascular disease (9, 11, 12). Moreover, removal of SA from cell surface has been demonstrated to accentuate low-density lipoprotein uptake into cells and thus lead to the pathogenesis of atherosclerotic plaques or thrombosis (13).

Despite the wide incorporation of SA in the cardiovascular system, and hence the potential impacts of dietary supplementation on the cardiovascular system, no studies on such dietary supplementation have been reported. It is in view of this, and the association of SAs in biological systems to lipid metabolism and coagulation system, we hypothesized that dietary supplementation may affect the overall flux of SA in the body with implications on diverse processes including lipid metabolism and the coagulation system. Positive findings in this regard could give added value in the discovery of anti-coagulation drugs. At present, there is increasing need for alternative therapies in view of side effects of currently available drugs (14). In this study, therefore, Neu5Ac was used instead of Neu5Gc, which is associated with harmful effects, to determine if its supplementation could modulate high fat diet (HFD)-induced hypercoagulation in rats.

## Materials and methods

### Materials

Neu5Ac (>98% purity and stable at room temperature) was purchased from Carbosynth Limited (Compton, Berkshire, UK) while simvastatin was purchased from Pfizer (New York, NY). Cholesterol was purchased from Amresco (Solon, OH) while cholic acid was purchased from Santa Cruz Biotechnology (Santa Cruz, CA). Lipid profile kits were purchased from Randox Laboratories Limited (Crumlin, County Antrim, UK). ELISA kits [Leptin, oxLDL, vWF (von Willebrand factor), prostacyclin, and thromboxane] were purchased from Elabscience Biotechnology Co., Ltd (Wuhan, China) while adiponectin was purchased from Millipore (Billerica, MA). RNA extraction kit was purchased from Bioscience Corp. (Taipei, Taiwan) while GenomeLab™ GeXP Start Kit was purchased from Beckman Coulter, Inc. (Miami, FL). RCL2 solution was purchased from Alphelys (Toulouse, France). Palm oil was purchased from Yee Lee Edible oils Sdn. Bhd. (Perak, Malaysia) while standard rat pellet was purchased from Specialty Feeds (Glen Forrest, WA).

### Animal handling and study

Animal ethical approval was obtained from the Animal Care and Use Committee (ACUC), Faculty of Medicine and Health Sciences, Universiti Putra Malaysia with approval number UPM/IACUC/AUP-R011/2014. Thirty 10-week-old Sprague Dawley male rats with body weights of 230–280 g were used as experimental animals. All rats were acclimatized for 2 weeks (25±2°C, 12/12 h light/dark cycle, ventilated air) with free access to clean pipe water and standard rat pellet. Rats were divided randomly into five different groups (*n=*6) as follows (Table 1): normal group (normal pellet), HFD group, simvastatin group (HFD+SIM, 10 mg/kg day simvastatin), low-dose Neu5Ac group [HFD+SAL (low-dose sialic acid), 50 mg/kg day], and high-dose Neu5Ac group [HFD+SAH (high-dose sialic acid), 400 mg/kg day Neu5Ac]. Neu5Ac was given to the rats by daily gavage after dissolving 900 mg in 10 ml water and sonication for 1 h. Body weights of rats were measured once weekly while food intake was measured daily. The rats were sacrificed after 12 weeks treatment. Serum samples and liver (preserved in RCL_2_) were collected accordingly and kept at −80°C for further analysis.

**Table 1:** Animal groups, food composition, food intake, and body weight

Animal group	Normal pellet (%)	Cholesterol/cholic acid (%)	Palm oil (%)	Starch (%)	Food intake (kcal/kg day)^a^^,^ b	Initial weight (g)^a^^,^ b	Final weight (g)^a^^,^ b
Normal	100.00	0.00	0.00	0.00	215.54±33.50	260.40±10.68	384.00±22.90
HFD	65.00	5.00	20.00	10.00	215.04±37.45	262.60±17.67	395.20±16.79
HFD+SIM	65.00	5.00	20.00	10.00	215.67±36.60	267.67±20.93	375.67±53.41
HFD+SAL	65.00	5.00	20.00	10.00	216.16±37.27	259.33±15.13	379.33±41.31
HFD+SAH	65.00	5.00	20.00	10.00	215.53±36.38	258.00±21.93	368.50±14.59

Normal pellet was acquired commercially while other diets were made in-house. HFD, high fat diet; SIM, simvastatin; SAL, low-dose sialic acid; SAH, high-dose sialic acid. HFD+SIM group rats gavage simvastatin 10 mg/kg day; HFD+SAL group rats gavage sialic acid 50 mg/kg day; HFD+SAH group rats gavage sialic acid 400 mg/kg day.

a Values are mean±SD (*n*=6);

b no statistical differences were observed between the groups in terms of food intake and weight changes.

### Biochemical and ELISA analysis

Lipid profile was analyzed using Randox analytical kits according to manufacturer's instructions using Selectra XL instrument (Vita Scientific, Dieren, The Netherlands). Serum samples were used in all relevant tests. Additionally, ELISA analyses were done according to kits’ protocol using serum samples. Absorbance readings were read on BioTeK Synergy H1 Hybrid Reader (BioTek Instruments, Inc., Winooski, VT) at the appropriate wavelengths.

### aPTT, PT, BT, PC, and RBC

For prothrombin time (PT) and activated partial thromboplastin time (aPTT) assays, blood was collected into trisodium citrated vacutainer tubes and analyzed using an automated coagulation analyzer according to the standard protocol (START-4, Diagnostica Stago, Parsippany, NJ). Prior to the measurement of bleeding time (BT), rats were anaesthetized with ketamine and xylazine (100 mg/kg/10 mg/kg) and placed on a hotplate which was heated up to 37°C. Then, 2 mm of the tail tip was amputated and the blood was blotted onto a filter paper every 30 s until no further blood stain was seen. The BT was calculated as the time needed for bleeding to stop after making a puncture wound on the rat tail. For hematologic cell counts, whole blood was collected in vacutainer tubes (K_3_EDTA) and analyzed using the MEDONIC CA530 hematology analyzer (Medonic, Stockholm, Sweden) within 2 h after blood collection.

### *Ex vivo* platelet aggregation

Effects of the interventions on *ex vivo* platelet aggregation were evaluated on baseline and endpoint blood samples using adenosine diphosphate (ADP) as agonist. Rat whole blood was collected into trisodium citrated vacutainer and centrifuged at 100×*g* for 20 min to obtain platelet-rich plasma (PRP). Then, 50 ng/mL of prostacyclin was mixed with the PRP to prevent platelet activation. Subsequently, the PRP was centrifuged at 240×*g* for 10 min to yield the platelet pellet. The platelet pellet was resuspended in Tyrode–HEPES buffer (134 mM NaCl, 2.9 mM KCl, 0.34 mM Na_2_HPO_4_, 12 mM NaHCO_3_, 20 mM HEPES, and 5 mM glucose, pH 7.3) and adjusted to a cell density of 10^8^ cells/mL. The solution (100 µL) was then mixed with 1 µL of ADP (to obtain a final concentration 10 µM) in a 96-well plate and shaken in double orbital mode throughout the whole analysis. The absorbance was read every 1 min for 20 min at 405 nm using BioTeK Synergy H1 Hybrid Reader (BioTek Instruments, Inc.).

### RNA extraction and gene expression

RNA was extracted from liver using RNA extraction kit. Nanospectrophotometer was used to measure the concentration and purity of all RNA samples. RNA samples were diluted to 20 ng/µL prior to gene expression study. Primers were designed online (www.ncbi.nlm.nih.gov/nucleotide/) using the *Rattus norvegicus* gene sequences. All the forward and reverse sequences were tagged with universal forward and universal reverse sequences (Table 2). Primers were purchased from Integrated DNA Technologies (Singapore city, Singapore) and reconstituted in RNAse free water. Reverse transcription and PCR were performed according to the GenomeLab™ GeXP Start Kit protocol (Beckman Coulter, Inc.). After RT and PCR, 1 µL of PCR product was analyzed on GeXP genomelab genetic analysis system (Beckman Coulter, Inc.) after mixing with sample loading solution and DNA size standard 400 according the manufacturer's instruction. The results were analyzed using Fragment Analysis module of the GeXP system software and normalization was done using eXpress Profiler software.

**Table 2:** Names, accession number, and primer sequences used in the study

Name	Left sequence	Right sequence
B2m^a^	AGGTGACACTATAGAATAATGCTTGCAGAGTTAAACA	GTACGACTCACTATAGGGATGCATAAAATATTTAAGGTAAGA
Hprt1^a^,^b^	AGGTGACACTATAGAATATCCTCATGGACTGATTATG	GTACGACTCACTATAGGGACTGGTCATTACAGTAGCTCTT
Rpl13a^a^	AGGTGACACTATAGAATAATGGGATCCCTCCAC	GTACGACTCACTATAGGGAATTTTCTTCTCCACATTCTT
Kan(r)^c^		
vWF	AGGTGACACTATAGAATAAGTACATTTGGGAGAAGAGG	GTACGACTCACTATAGGGACTCTTCCGACTTACAATCTC
Adipoq	AGGTGACACTATAGAATACCAAAAGTTCCAGGACTC	GTACGACTCACTATAGGGAGGTCACCCTTAGGACCA
Leptin	AGGTGACACTATAGAATACAAGTATCCCTGGTAGATGT	GTACGACTCACTATAGGGAAAGTTTTCTTGCATTGATCT
PAI-1	AGGTGACACTATAGAATAACAAGTCTTTCCGACCA	GTACGACTCACTATAGGGAAGAGGATTGTCTCTGTTGG

RT conditions were: 48°C for 1 min; 37°C for 5 min; 42°C for 60 min; 95°C for 5 min, then hold at 4°C. PCR conditions were initial denaturation at 95°C for 10 min, followed by two-step cycles of 94°C for 30 sec and 55°C for 30 sec, ending in a single extension cycle of 68°C for 1 min.

a Housekeeping genes;

b normalization gene. Underlined sequences are universal left and right sequences (tags);

c internal control supplied by Beckman Coulter, Inc. as part of the GeXP kit.

### Data analysis

Quantitative data were expressed in means±standard deviation and calculated using one-way analysis of variance (ANOVA) with Tukey's multiple comparison test to compare the difference among different groups using SPSS 17.0 (SPSS, Inc., Chicago, IL). *p*<0.05 was considered statistically significant.

## Results and discussion

### Lipid profile analysis

After 12 weeks intervention, the normal group had better lipid profile compared to the other groups fed with HFD (Table 3). The HFD group had significantly higher triglyceride level compared with the rest of the groups, while no significant differences were observed between the normal, HFD+SIM, HFD+SAL, and HFD+SAH groups. This possibly indicated a triglyceride-lowering property by Neu5Ac, similar to the effect of simvastatin. Additionally, the HFD+SIM group reduced the total cholesterol level compared with the other groups that also received HFD. No significant differences were observed for LDL and HDL between all the groups that received HFD. Although the HFD+SIM group had better lipid profiles than the other HFD-fed groups, the TG/HDL was not significantly different, while the HFD+SAL and HFD+SAH groups showed differences in triglyceride and TG/HDL. TG/HDL is an important predictor for cardiovascular disease that is suggested to be a more potent indicator than TG or HDL alone (15). The overall results suggested that dietary supplementation with Neu5AC could improve lipid profiles, which may have been mediated through the differential sialylation of glycoprotein structures across the body as a result of increased Neu5Ac flux within the body (16).

**Table 3:** Biochemical parameters

Rat groups	Chol. (mmol/L)	Trig. (mmol/L)	LDL (mmol/L)	HDL (mmol/L)	LDL/HDL	TG/HDL
Normal	1.55±0.43	0.62±0.15	0.28±0.11	1.18±0.35	0.24±0.04	0.55±0.15
HFD	7.47±1.13^a^	1.21±0.38^a^	4.98±1.03^a^	1.05±0.13	4.77±0.98^a^	1.16±0.33^a^
HFD+SIM	4.99±1.11^a^^,^ b	0.63±0.18^b^	3.6±1.1^a^	1.04±0.17	3.46±0.94^a^	0.62±0.22
HFD+SAL	5.68±2.18^a^	0.54±0.07^b^	4.48±1.81^a^	1.04±0.28	4.14±0.79^a^	0.54±0.09^b^
HFD+SAH	5.05±2.07^a^	0.54±0.07^b^	3.67±1.58^a^	1.08±0.27	3.31±1.08^a^	0.52±0.08^b^

Data represent mean±SD (*n*=6). HFD, high fat diet; SIM, simvastatin; SAL, low-dose sialic acid; SAH, high-dose sialic acid; HFD+SIM group rats gavage simvastatin 10 mg/kg day; HFD+SAL group rats gavage sialic acid 50 mg/kg day; HFD+SAH group rats gavage sialic acid 400 mg/kg day; HDL, high-density lipoprotein; LDL, low-density lipoprotein; Chol, cholesterol; SIM, simvastatin; Trig, triacylglycerol.

a Statistical difference in comparison with the normal group (*p*<0.05) according to Tukey's multiple comparison test;

b statistical difference in comparison with the HFD group (*p*<0.05) according to Tukey's multiple comparison test.

### aPTT, PT, BT, PC, and RBC and *ex vivo* platelet aggregation

**Fig. 1:**
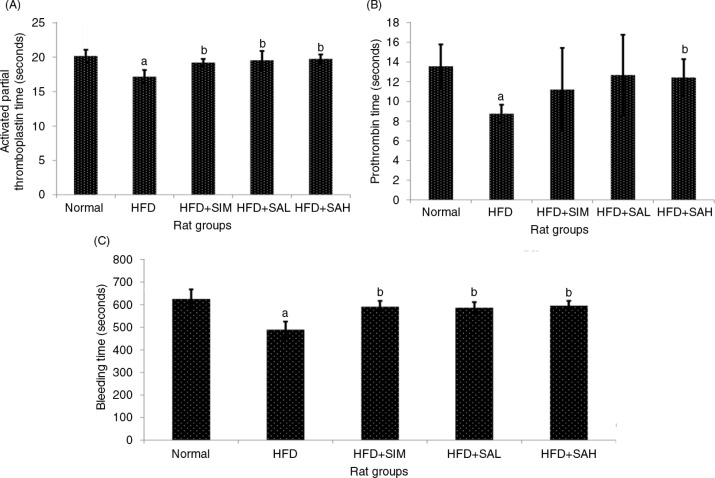
Effects of sialic acid (SA) on (A) activated partial thromboplastin time (APTT), (B) prothrombin time (PT), and (C) bleeding time (BT) in high fat diet (HFD)-fed rats. Values are mean±SD (*n*=6). Groups are similar to those in Table 1. ^a^Statistical difference in comparison with the normal group (*p*<0.05) according to Tukey's multiple comparison test; ^b^statistical difference in comparison with the HFD group (*p*<0.05) according to Tukey's multiple comparison test.

aPTT and PT are important parameters of the intrinsic and extrinsic coagulation pathways (17). Figure 1A shows that aPTT was significantly shorter in the HFD group compared with the other groups (*p=*0.002 for normal, and *p=*0.026 for both HFD+SAL and HFD+SAH groups) except the HFD+SIM group (*p*=0.10). A similar pattern was observed for PT (Fig. 1B). The HFD+SAH group had similar aPTT and PT values to the normal group, which were significantly different in comparison with the HFD group (*p*=0.026 and 0.002 for aPTT, *p*=0.02 and 0.003 for PT, respectively). Furthermore, BT showed a similar trend with aPTT; the BT of the HFD group was significantly shorter compared with the other groups (*p*<0.001), (Fig. 1C). These results indicated that HFD may promote the risk of hypercoagulation similar to what is reported in hyperlipidemic conditions (18), while Neu5Ac attenuated the HFD-induced procoagulant effects.

**Fig. 2:**
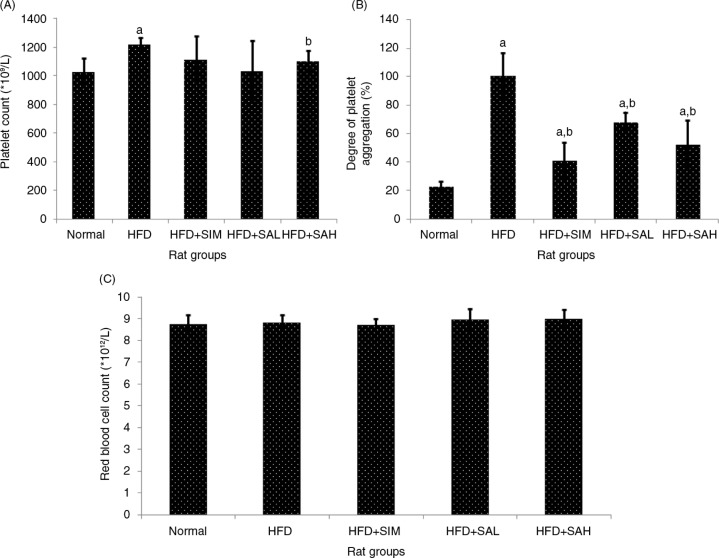
Effects of sialic acid (SA) on (A) platelet count, (B) platelet aggregation, (C) red blood cell (RBC) count in high fat diet (HFD)-fed rats. Values are mean±SD (*n*=6). Groups are similar to in Table 1. ^a^Statistical difference in comparison with the normal group (*p*<0.05) according to Tukey's multiple comparison test; ^b^statistical difference in comparison with the HFD group (*p<*0.05) according to Tukey's multiple comparison test.

The platelet count (PC) was higher in the HFD group in comparison with other groups (*p=*0.002), especially in the normal (*p*=0.001) and HFD+SAH group (*p*=0.042) (Fig. 2A), which had significantly higher platelet aggregation compared with the other groups (*p*<0.001) (Fig. 2B). Increased PC and platelet aggregation have been linked with hypercoagulation (19, 20), and the results, therefore, corroborate those of the aPTT, PT, and BT. With regard to RBC count, no differences were observed between all the groups (*p*>0.05; Fig. 2C). Overall, the results showed that HFD promoted hypercoagulation likely due to hyperlipidemia (18), while Neu5Ac supplementation attenuated the hypercoagulation associated with HFD-induced hyperlipidemia. In addition to the effect of Neu5Ac on ameliorating hyperlipidemia, its increased flux could have influenced the coagulation system to reduce the HFD-induced coagulation (16). Moreover, Neu5Ac is present on the vascular endothelium and erythrocyte, and its increased levels on these surfaces will increase the negative charge that prevents blood clotting (11, 12, 16).

### Serum oxLDL, vWF, leptin, thromboxane, adiponectin, and prostacyclin

**Fig. 3:**
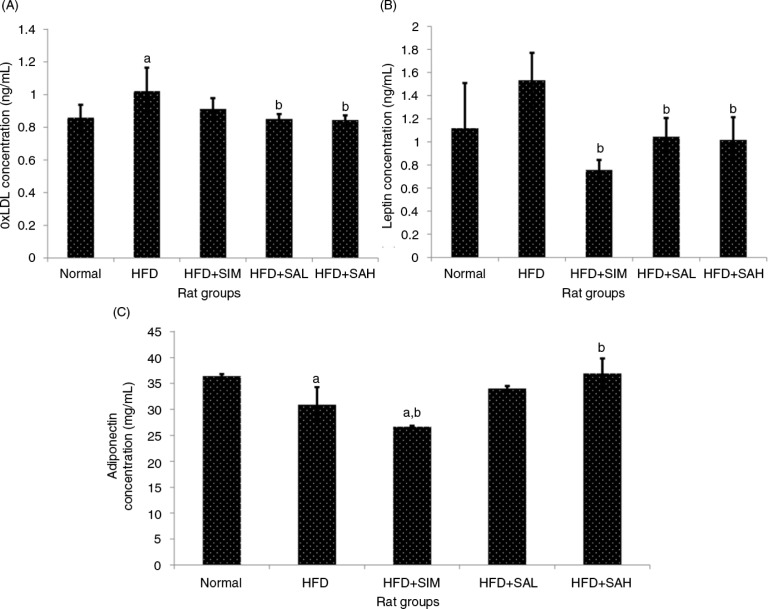
Effects of sialic acid (SA) on (A) serum oxLDL, (B) serum leptin, and (C) serum adiponectin in high fat diet (HFD)-fed rats. Values are mean±SD (*n*=6). Groups are similar to in Table 1. ^a^Statistical difference in comparison with the normal group (*p*<0.05) according to Tukey's multiple comparison test; ^b^statistical difference in comparison with the HFD group (*p*<0.05) according to Tukey's multiple comparison test.

**Fig. 4:**
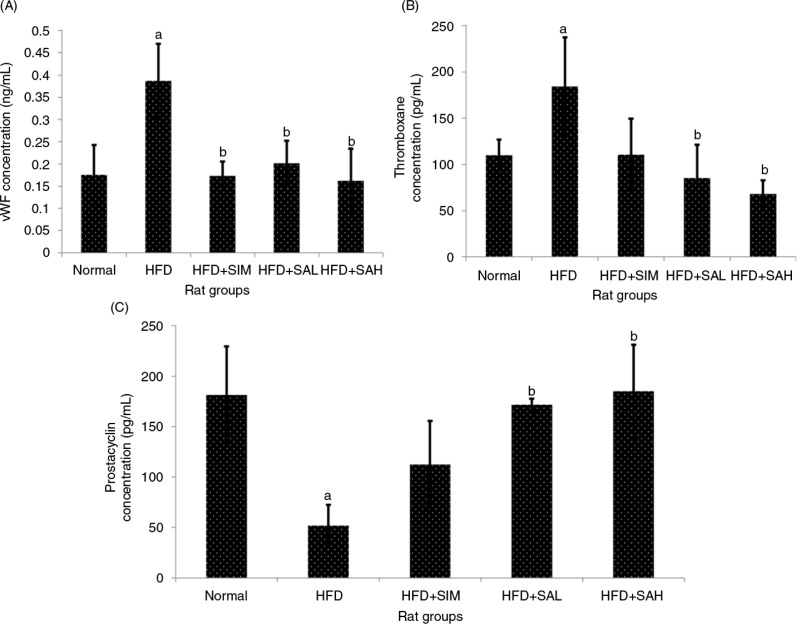
Effects of sialic acid (SA) on (A) serum vWF, (B) serum thromboxane, and (C) serum prostacyclin, in high fat diet (HFD)-fed rats. Values are mean±SD (*n*=6). Groups are similar to those in Table 1. ^a^Statistical difference in comparison with the normal group (*p*<0.05) according to Tukey's multiple comparison test; ^b^statistical difference in comparison with the HFD group (*p*<0.05) according to Tukey's multiple comparison test.

Increased levels of leptin, oxidized low-density lipoprotein (oxLDL), vWF, and thromboxane, and decreased levels of adiponectin and prostacyclin are linked to a heightened risk of cardiovascular disease (21–24). In the present study, the patterns of these markers suggested an increased risk of cardiovascular disease while Neu5Ac attenuated the risk (Figs. 3 and 4); ox-LDL was lower for the normal, HFD+SAH, and HFD+SAL groups compared with the HFD group (*p*=0.048, 0.028, and 0.037, respectively), while adiponectin was higher for the normal and HFD+SAH groups (*p*=0.038 and 0.001, respectively) but not the HFD+SAL group (*p*=0.096) in comparison with the HFD group. The normal and HFD+SIM groups also had significantly higher and lower adiponectin levels compared with the HFD group (*p*=0.038 and 0.044), respectively. The HFD+SIM, HFD+SAL, and HFD+SAH groups had lower leptin levels compared to the HFD group (*p*=0.001, 0.041, and 0.029, respectively). Neu5Ac increased adiponectin levels while simvastatin did not, suggesting that simvastatin treatment may have negative implications for the cardiovascular system as reported previously (25). vWF was also lower in the HFD+SAH and HFD+SAL groups compared with the HFD group (vWF, *p*<0.001 and *p*=0.002, respectively), while thromboxane was only lower in the HFD+SAH group (*p=*0.04). Prostacyclin on the other hand was higher in both groups compared with the HFD groups (*p*<0.001 and *p*=0.001, respectively). The results of vWF, thromboxane, and prostacyclin for the normal group were similar to those of the HFD+SAH group. From the results obtained, it was apparent that the HFD+SAH, HFD+SAL, and HFD+SIM groups had similar effects on vWF but the results were better in the case of prostacyclin and thromboxane in the Neu5Ac-treated groups. As can be recalled, SA present on the surfaces of vascular walls and erythrocytes regulates the uptake of low-density lipoprotein by the endothelium and erythrocytes, respectively, and possibly its oxidation which prevents thrombosis (13, 26). The presence of increased concentrations of Neu5Ac on vascular walls and erythrocytes due to its supplementation in this study may also have prevented the oxidative changes on LDL from taking place, thereby regulating further inflammation and hence the improved coagulation markers (11, 16, 26).

### Hepatic mRNA level of leptin, adiponectin, and coagulation-related genes

**Fig. 5:**
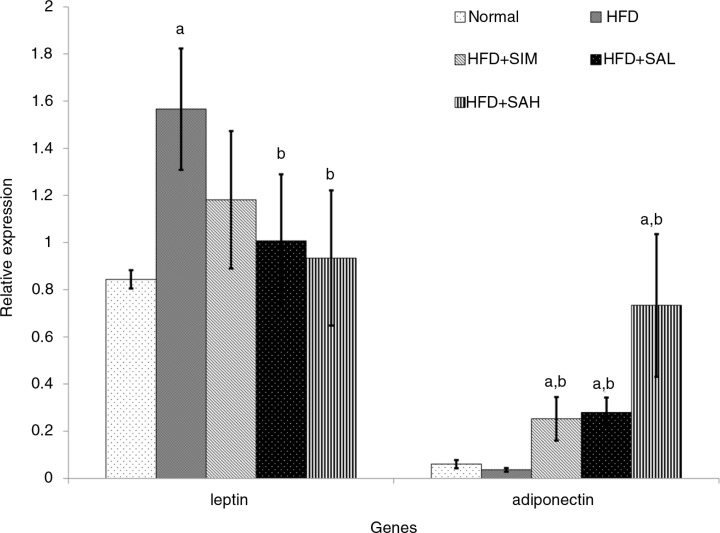
Effects of sialic acid (SA) on the mRNA levels of hepatic leptin and adiponectin (adipoq) genes in high fat diet (HFD)-fed rats. Values are mean±SD (*n=*6). Groups are similar to in Table 1. ^a^Statistical difference in comparison with the normal group (*p<*0.05) according to Tukey's multiple comparison test; ^b^statistical difference in comparison with the HFD group (*p*<0.05) according to Tukey's multiple comparison test.

**Fig. 6:**
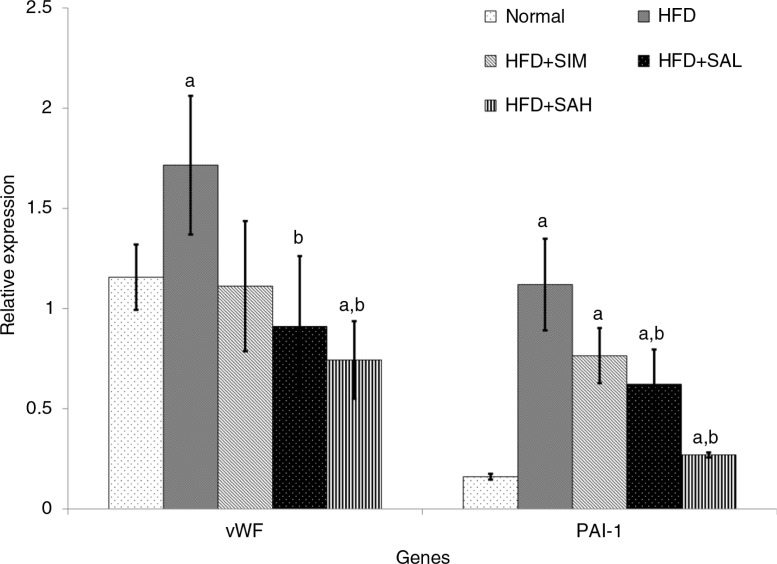
Effects of sialic acid (SA) on the mRNA levels of hepatic von Willebrand factor (vWF) and plasminogen activator inhibitor-1 (PAI-1) genes in high fat diet (HFD)-fed rats. Values are mean±SD (*n*=6). Groups are similar to those in Table 1. ^a^Statistical difference in comparison with the normal group (*p<*0.05) according to Tukey's multiple comparison test; ^b^statistical difference in comparison with the HFD group (*p*<0.05) according to Tukey's multiple comparison test.

Figures 5 and 6 show the effects of the different interventions on the mRNA levels of hepatic metabolic and coagulation-related genes. The HFD+SIM, HFD+SAL, and HFD+SAH groups downregulated the leptin gene expression in comparison with the HFD group, although only the Neu5Ac groups showed significantly lower expression levels (*p*=0.04 and 0.042 for HFD+SAL and HFD+SAH group, respectively). Hepatic adiponectin expression was low in both normal and HFD groups, while the Neu5Ac-treated groups had the highest levels. The differences in hepatic expression and serum levels of adiponectin in the simvastatin group may be due to some post-transcriptional modifications that produced low levels of the adiponectin protein (27). Both the HFD+SIM and Neu5AC-treated groups improved the adiponectin expression compared with the HFD group (*p*=0.044, 0.096, and 0.001 for HFD+SIM, HFD+SAL, and HFD+SAH groups, respectively), with a dose-dependent trend in the case of the Neu5AC-treated groups. Furthermore, hepatic expression of coagulation-related genes (vWF and PAI-1) was reduced more than in the HFD+SIM group and significantly lower than that of the HFD group [vWF, *p*=0.077, 0.006, and 0.003 for HFD+SIM, HFD+SAL, and HFD+SAH groups, respectively; plasminogen activator inhibitor-1 (PAI-1), *p*=0.083, 0.027, and 0.001 for HFD+SIM, HFD+SAL, and HFD+SAH groups, respectively].

SAs have been linked with diverse biological functions, which are related to their terminal positions on the surfaces of cells (28). Their negative charges can regulate intermolecular and intercellular interactions and stabilize different proteins including enzymes for effective functioning (29). These interactions have implications on the overall biochemistry in the body, and in particular have been shown to regulate the levels of coagulation and possibly inflammation in the cardiovascular system (11, 12, 16). Moreover, SAs present on the surfaces of blood cells can influence the quality of these cells and by extension the functioning of the cardiovascular system through the controlled degradation of the blood cells (29). Additionally, amyloid beta in the brains of Alzheimer's disease patients has been shown to develop mostly at the sites of abnormally clustered SAs, in contrast with soluble SA that can attenuate the amyloid beta-induced toxicity on neuronal cells (30). Similarly, the recognition that SA present in human milk enhances cognitive development in infants has formed the basis for the addition of SA in formula milk (31). Moreover, the brain has the highest concentration of SAs in the human body, and it may be the most important site of action of these molecules (32). Studies have also shown that cell surface SAs can regulate the colonization of host cells by microbes thereby preventing infection. They also help the immune system cells to distinguish between body cells and foreign cells (29, 32). Despite the biological importance of SAs, only limited studies have demonstrated their nutritional significance as supplements, similar to what we have demonstrated in the present study. It can be argued that the ability of SA to modulate a wide variety of biological functions and the effects of SA flux on the membrane-bound SA molecules suggest that its supplementation can influence many processes in the body. This hypothesis is supported by the data in the present study, which shows that SA can modulate lipid profiles and coagulation in rats.

In total, we have demonstrated that HFD feeding caused dyslipidemia and hypercoagulation in rats, which were mediated through transcriptional regulation of related hepatic genes. On the other hand, Neu5Ac and simvastatin treatments attenuated the HFD-induced changes, although Neu5Ac produced better effects. Transcriptional changes are often the earliest changes during metabolic perturbations, which later result in detectable biochemical changes, and there are suggestions that treatments that can regulate such transcriptional changes can have longer lasting effects than non-transcriptionally mediated effects (33). Interestingly, simvastatin, which is a standard drug in the management of hyperlipidemia showed a tendency for worsening metabolism, as indicated by its effects on serum adiponectin levels. Moreover, simvastatin has been reported to increase the risk of metabolic disorders like type 2 diabetes (34), which may be connected to its effects on adiponectin (25). The effects of Neu5Ac observed in this study could have been mediated through increased concentrations of the Neu5Ac in the vascular walls and erythrocyte due to its increased flux in the body, which resulted in increased negative charges on these surfacing thereby regulating the interaction between biomolecules like LDL and the vascular wall, with consequently reduced inflammation and coagulation.

## Conclusions

In the present study, we successfully demonstrated that Neu5Ac prevented HFD-induced hypercholesterolemia and hypercoagulation through regulation of serum lipid levels, metabolic indices, and coagulation-related indices. These effects of Neu5Ac were partly mediated at the transcriptional level. Considering the complex nature of SA metabolism, there is the need to further evaluate the exact mechanism by which dietary SA produces its effects. Long-term studies including clinical trials are also indicated to determine the clinical validity of the present findings. The results from these studies may pave the way for the use of Neu5Ac in the prevention of hyperlipidemia-induced coagulation-related cardiovascular events, and are worth studying further.
